# Supplementary material for: Native elongation transcript sequencing reveals temperature dependent dynamics of nascent RNAPII transcription in Arabidopsis
Source: Nucleic Acids Res. 2019 Dec 21;48(5):2332–47. doi: 10.1093/nar/gkz1189 (PMC7049701; doi:10.1093/nar/gkz1189)
Supplement: gkz1189_Supplemental_Files [file gkz1189_supplemental_files.zip › Supplementary Figs 1-8.pdf]

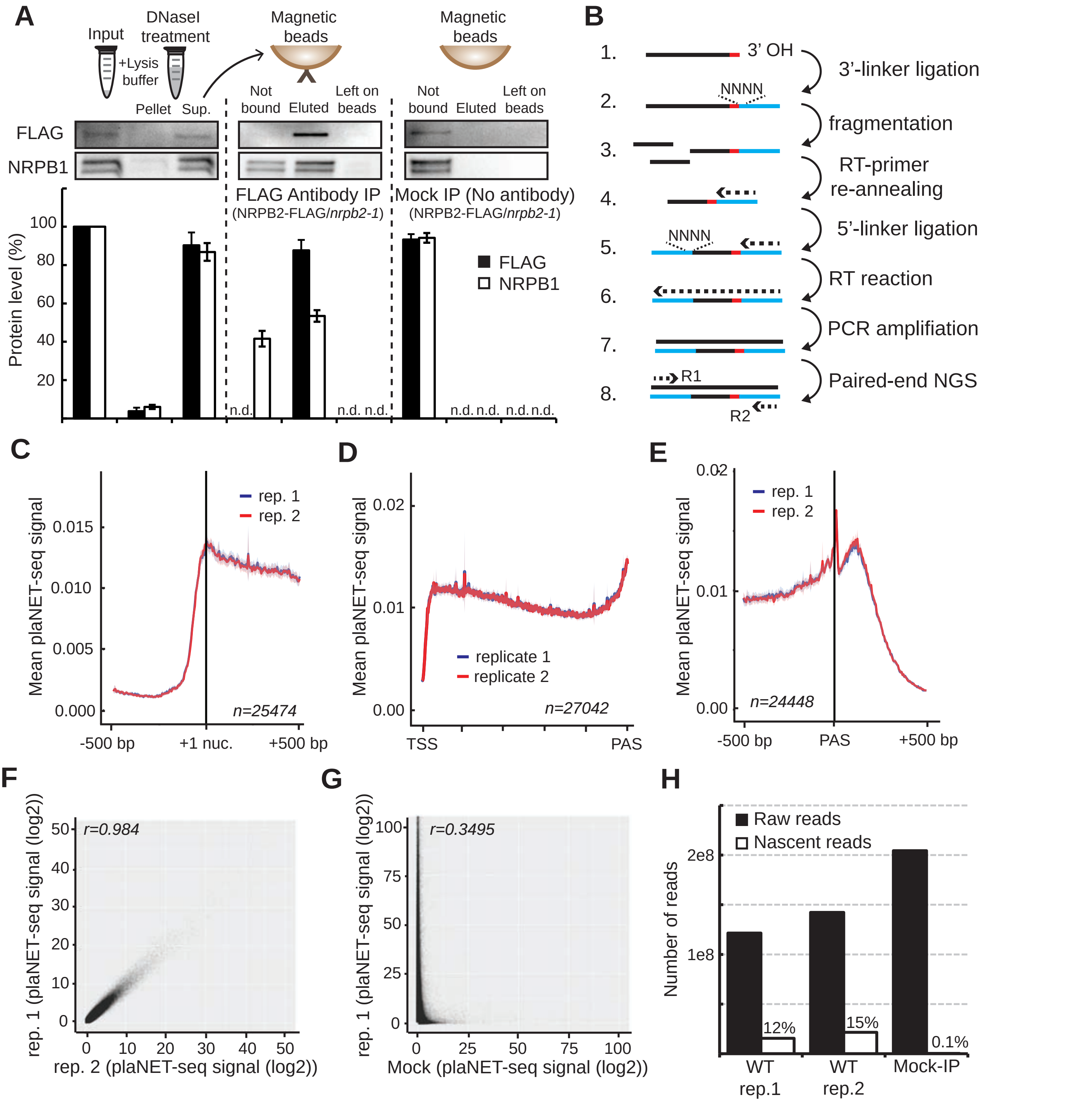

Supplementary Figure 1:

A, Western blots of NRPB2-FLAG and NRPB1 during the immunoprecipitation and elution steps of the plaNET-seq protocol. Upper panel shows representative blots (repeated with at least 3 biological replicates). Lower panel shows quantifications of proteins levels from blots. n.d. denotes non-detectable levels. Purification and elution of RNAPII complexes were efficient. A mock-IP showed no FLAG or NRPB1 signal in the elution, indicating high stringency of the purification.
B, Flow chart of the construction of plaNET-seq libraries. A 3'-linker was first ligated to the 3'OH (last base added by RNAPII, red dot) of nascent RNAs followed by alkaline fragmentation. Subsequently, a 5'-linker was ligated followed by an RT-reaction. A PCR reaction with barcode primers was performed before deep-sequencing. Both the 3'- and 5'-adapter contained 4 randomized bases that decreased the sequence bias of the RNA ligase and allowed for removal of PCR duplicates.
C-E, Metagene analyses of the plaNET-Seq signal anchored at the center of the first nucleosome (c) over whole genes with length 0.5-5 Kb (d) or anchored at the Poly(A)-site (e). Two biological replicates of untreated wild type sample are represented by blue (replicate 1) and red (replicate 2). The two replicates show very high reproducibility of metagene profiles. The shaded area shows 95% confidence interval for the mean.
F, Scatter plot of the reproducibility of plaNET-seq libraries. Correlation coefficient was determined using the Pearson method.
G, Scatter plot of the WT (rep. 1) and Mock libraries. Correlation coefficient was determined using the Pearson method.
H, Bar chart showing raw and nascent reads (raw reads without PCR duplicates and contaminant reads) in two WT replicates and a mock-IP sample. The numbers denote the percentage of nascent reads compared to raw reads for each library. The mock-IP sample showed extremely low level of nascent reads (0.1% compared to 12-15% for WT replicates).

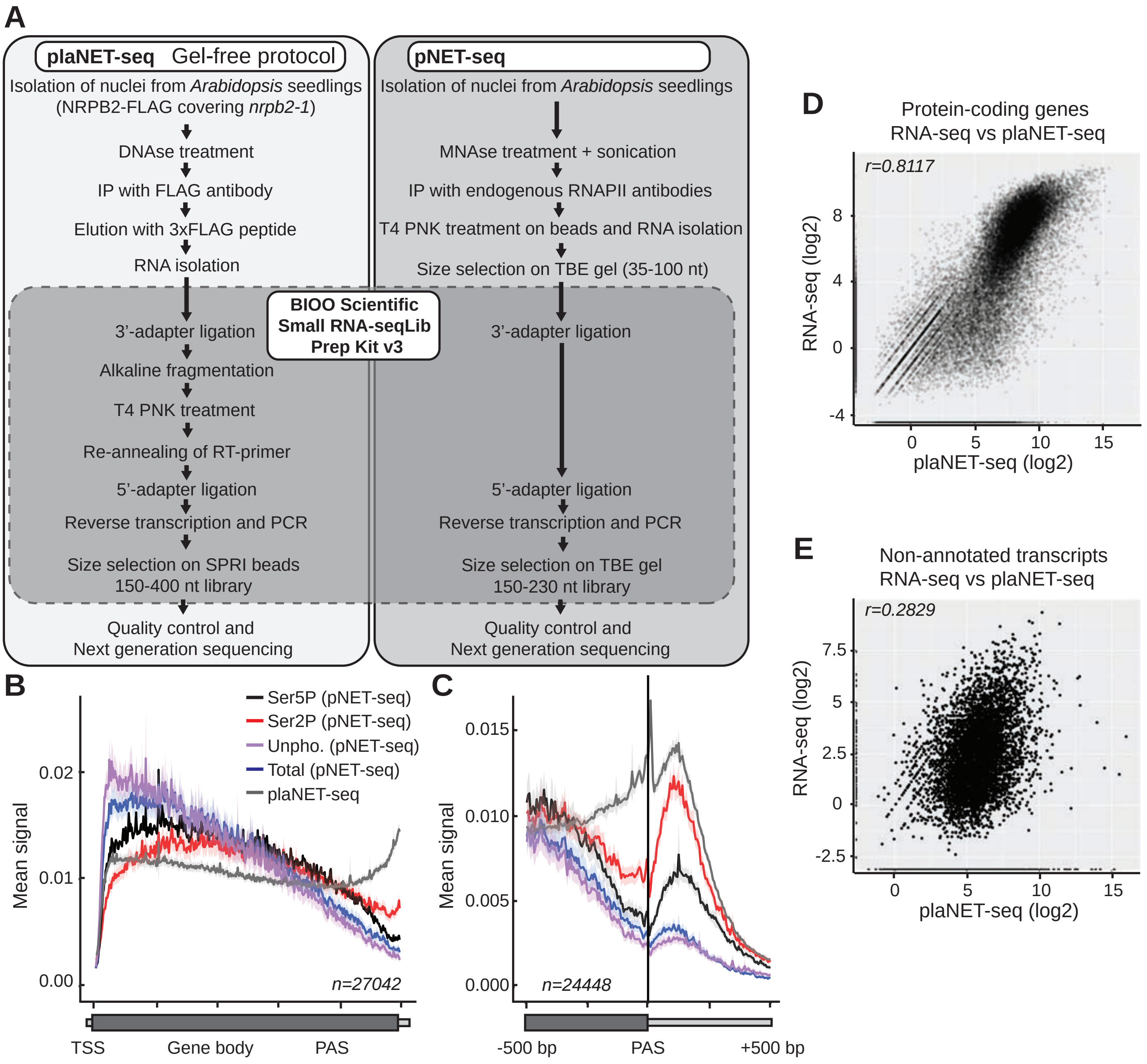

Supplementary Figure 2:
A, Flowchart over the plaNET-seq and pNET-seq protocols. plaNET-seq offers a gel-free protocol that starts with isolated nuclei from a NRPB2-FLAG/nrpb2-1 line. Nuclei are lysed together with DNase I followed by FLAG-IP. RNAPII complexes are eluted competitively with a 3xFLAG peptide followed by RNA isolation. pNET-seq uses MNase and sonication to lyse nuclei followed by IP with endogenous RNAPII antibodies. The isolated RNA is run on a gel and size selected by cutting the gel. Both protocols use the NEXTflex Small RNA-seq Kit version 3. pNET-seq follows the instructions of the manufacturers manual for generation of libraries while plaNET-seq adds the 3'-linker before a fragmentation step. In addition, plaNET-seq does not include any extra size selection other than the SPRI beads used in the kit.
B-C, Metagene analyses of the plaNET-seq signal over whole genes with length 0.5-5 Kb (b) or anchored at the Poly(A)-site (c). Data from the Ser5P antibody is shown in black, Ser2P in red, Unphosphorylated in purple and total RNAPII in blue. plaNET-seq data is shown in grey. The shaded area shows 95% confidence interval for the mean.
D, Scatter plot of the expression level of protein-coding transcripts as determined by strand-specific RNA-seq and plaNET-seq. Correlation coefficient was determined using the Spearman method. The correlation was high for protein-coding transcripts (i.e. mRNA).
E, Scatter plot of the expression level of non-annotated transcripts comparing strand-specific RNA-seq and plaNET-seq. Correlation coefficient was determined using the Spearman method. The correlation was low for non-annotated transcripts (i.e. lncRNA).

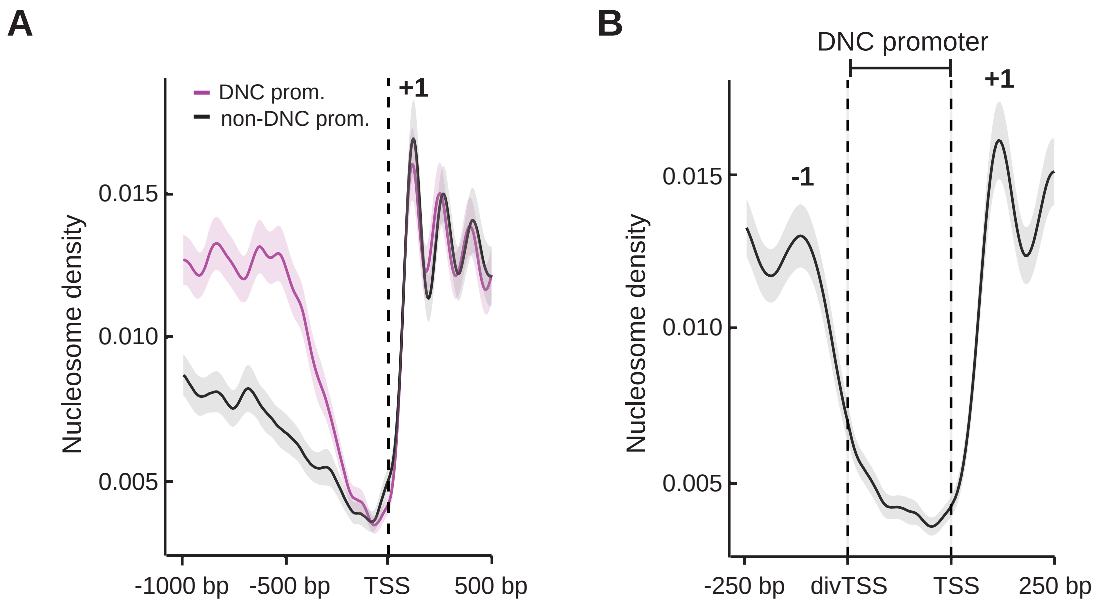

Supplementary Figure 3:
A, Metagene analysis of nucleosome density (DNC promoters versus non-DNC control promoters) in 1.5 Kb windows anchored at the sense TSS. The control promoters were chosen to match the DNC promoters by transcription level on the sense strand. The shaded area shows 95% confidence interval for the mean.
B, Metagene analysis of nucleosome density (DNC promoters only) in variable width windows anchored at both the sense TSS and at the divTSS (with 250 bp fixed width flanks). The shaded area shows 95% confidence interval for the mean.

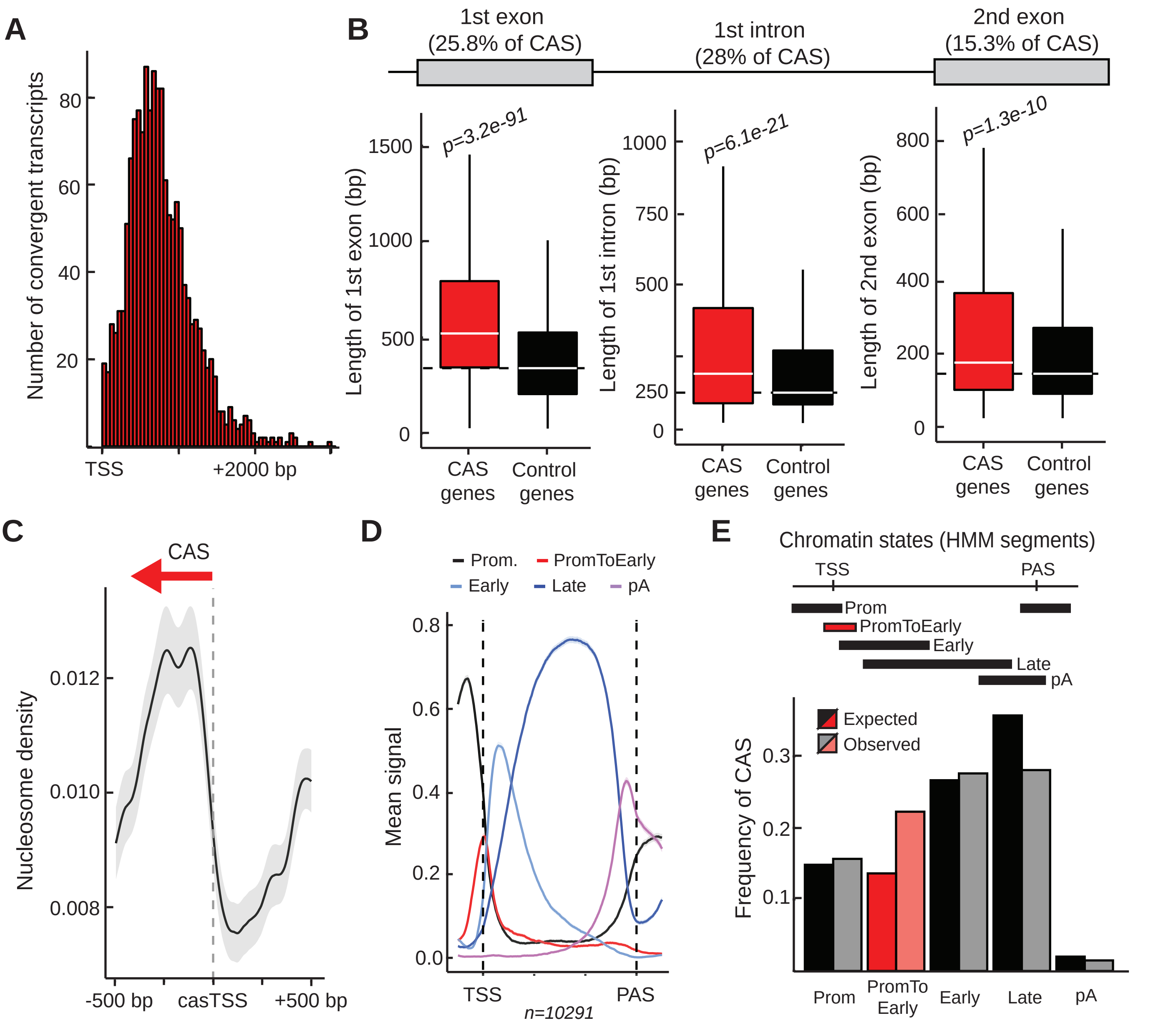

Supplementary Figure 4:

A, Histogram of absolute distance (bp) between the start site of convergent antisense transcripts (casTSS) and the sense TSS. CAS transcription tended to initiate within the first 1000 bp from the sense TSS.

B, Box plots of the length of the first exon (left), first intron (middle) and second exon (right) of genes with a CAS and without a CAS. Percentage denotes how many CAS start within each feature. CAS host genes tend to have longer exons and introns in their 5'-end.

C, Metagene analysis of nucleosome density in 1 kb windows centered at the convergent transcript start site (casTSS). The shaded area shows 95% confidence interval for the mean.

D, The distribution of chromatin state groups along nuclear protein coding genes (FPKM ≥ 1, length 1-5 Kb). The metagene plot covers the gene body (scaled to 300 bins) and includes 250 bp flanks upstream and downstream of TSS and PAS, respectively. The following groups of chromatin states from the PCSD database were defined: Promoter (Prom; states 13, 15-21), promoter-to-early elongation (PromToEarly; states 22-23), early elongation (Early; states 24-26), late elongation (Late; states 3-12, 27-28) and termination (pA; states 1-2).

E, Metagene analysis of chromatin states determined by ChromHMM along the gene bodies of Arabidopsis genes. Based on the PCSD database, the following states were assigned to respective group: promoter (Prom; states 13, 15-21), promoter-to-early elongation (PromToEarly; states 22-23), early elongation (Early; states 24-26), late elongation (Late; states 3-12, 27-28) and polyA (pA; states 1-2). Each CAS was assigned a chromatin state group based on overlap with casTSS. Observed frequencies of casTSS were plotted together with the expected frequencies of overlap based on the random model.

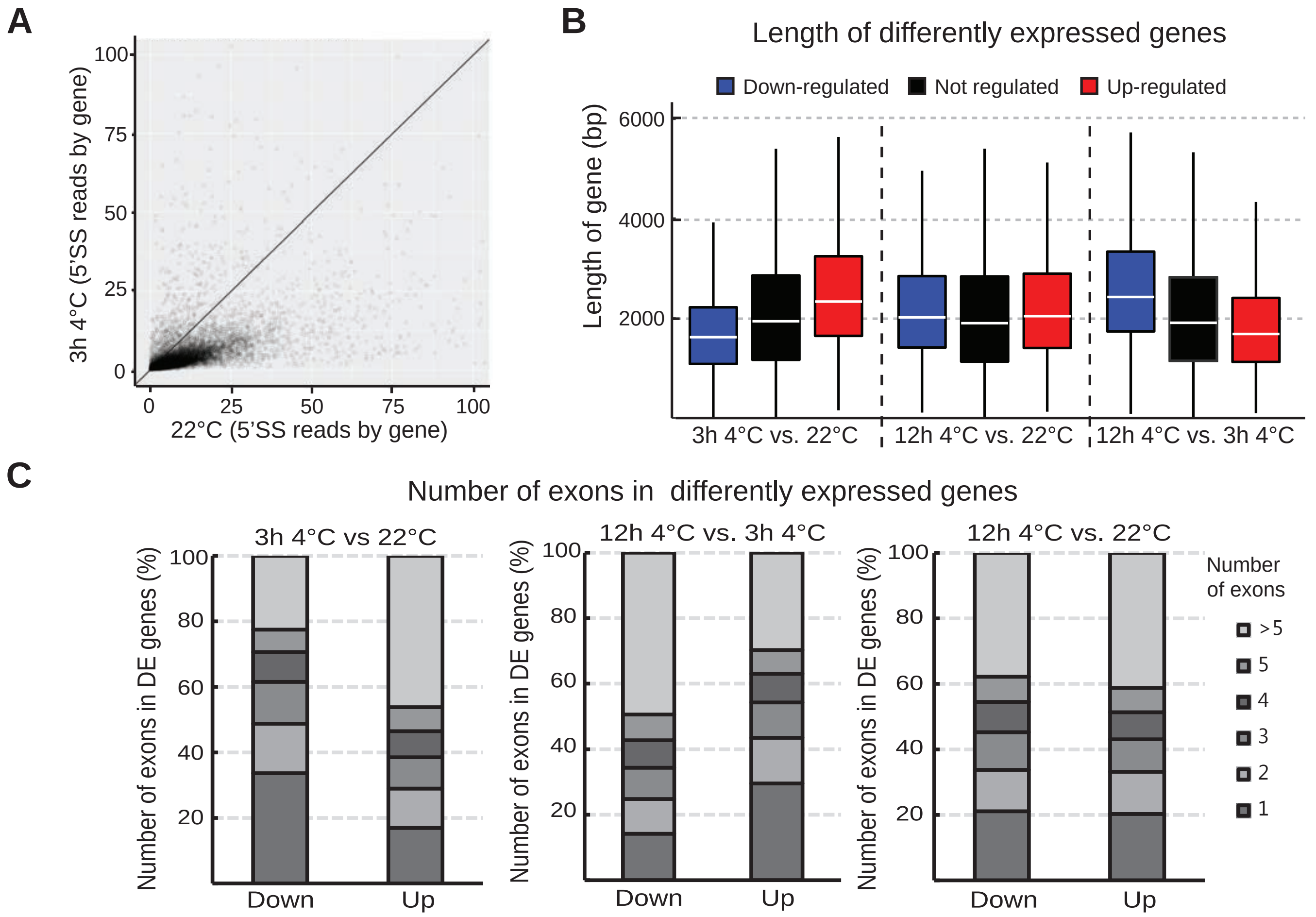

Supplementary Figure 5:
A, Scatter plot of the 5' splice site (5'SS) signal in 22°C compared to 3h at 4°C determined by plaNET-seq. The vast majority of 5'SS have less spliceosomal reads after 3h at cold than at 22°C.
B, Box plots of the gene length in bp grouped by their differentially transcribed status in response to low temperature (as determined by plaNET-Seq). The plot shows genes which are differentially transcribed between 3h 4°C and 22°C (left), 12h 4°C and 22°C (middle) and 12h 4°C and 3h 4°C (right). Down-regulated genes are shown in blue, non-regulated in black and up-regulated genes in red. The plot shows that genes down-regulated after 3h at 4^{0}C tend to be shorter while up-regulated genes are longer. The opposite trend was detected between 12h at 4°C and 3h at 4°C. Here, down-regulated genes tend to be long while up-regulated genes are short.
C, Bar chart of the number of exons in genes differentially transcribed between 12h 4°C and 0h 4°C. Both down- and up-regulated genes show similar number of exons.

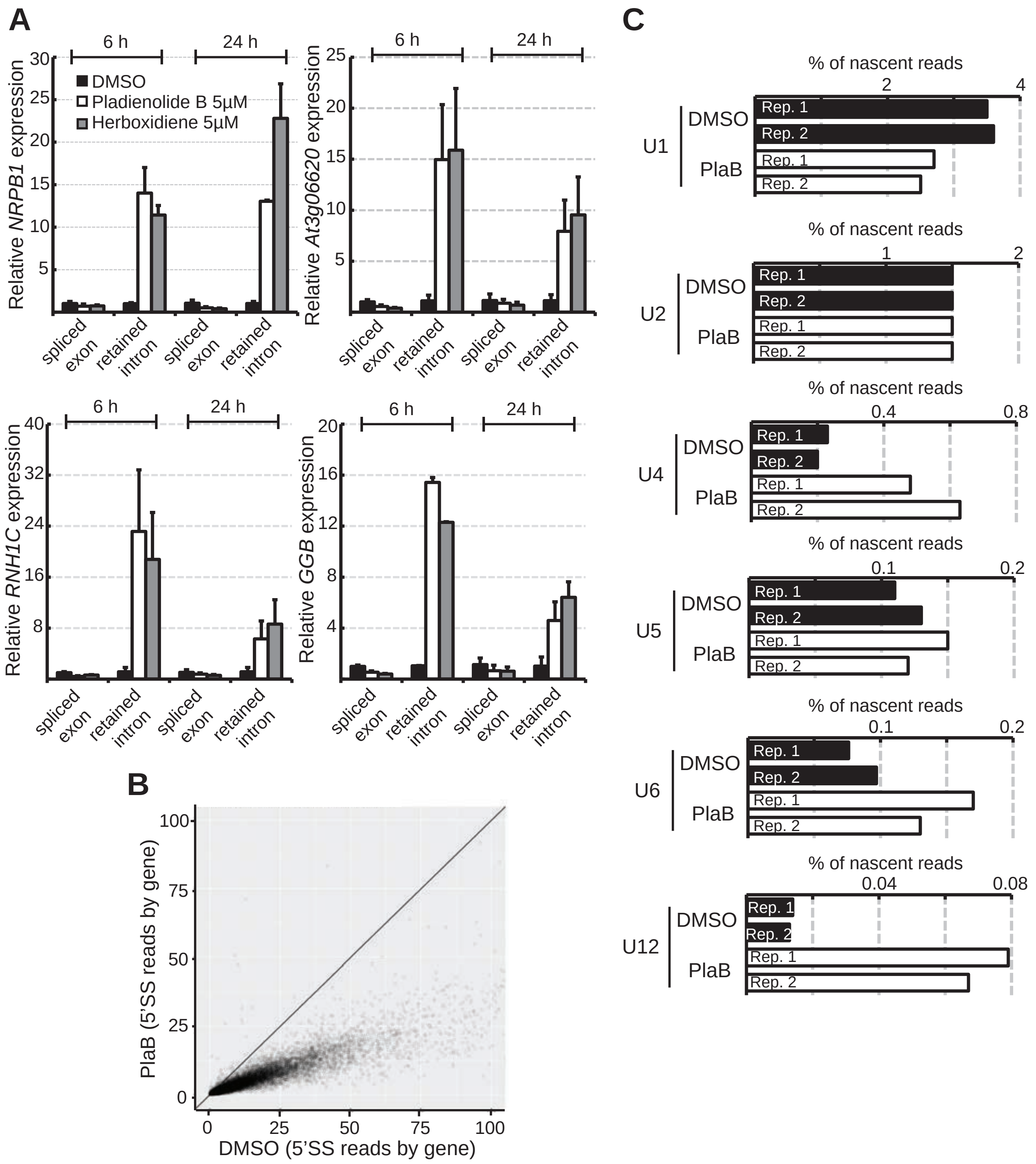

Supplementary Figure 6:

A, RT-qPCR confirmation of the plaB and Herboxidiene treatment (6h and 24h) shown for different splicing events (At4g35800, At3g06620, At1g24090 and At2g39550). Seedlings treated with DMSO for the same time were used as control. Bars represent mean ± SEM of three biological replicates.

B, Scatter plot of the 5' splice site (5'SS) signal in DMSO compared to 6h of PlaB treatment determined by plaNET-seq. The vast majority of 5'SS have less spliceosomal reads after PlaB treatment.

C, Bar chart of the percentage of processed reads that aligned to different families of small nuclear RNAs involved in splicing. plaNET-seq DMSO and plaB replicates are shown.

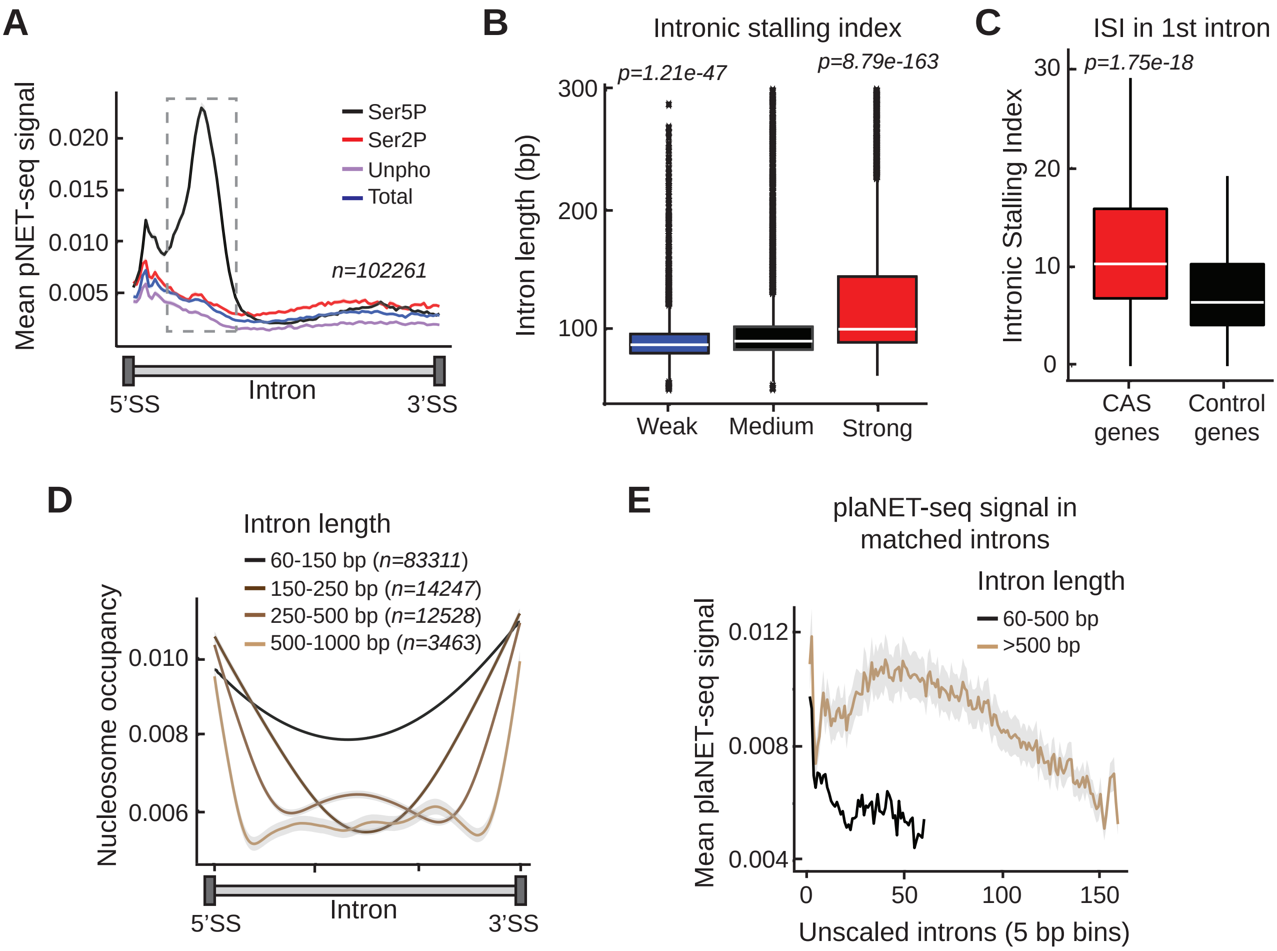

Supplementary Figure 7:

A, Metagene analysis of nascent RNAPII transcription in introns (with length 50-300 bp) as determined by pNET-seq. Data from the Ser5P antibody is shown in black, Ser2P in red, Unphosphorylated in purple and total RNAPII in blue. Dashed box indicates stalling site at the 3'-end of exons. The shaded area shows 95% confidence interval for the mean.

B, Box plots of intron length grouped by their intronic stalling index (ISI). Statistical significance of differences was measured by two-sided Mann-Whitney U test. Introns with strong intronic stalling tended to be longer and introns with weak stalling shorter compared to introns with medium stalling.

C, Box plot of Intronic Stalling Index (ISI) for the first intron of genes harboring a CAS. First introns of CAS host genes tend to have a stronger ISI compared to control genes.

D, Metagene analysis of nucleosome occupancy in introns stratified by their lengths. Introns were scaled to 300 bins. Short introns (60-250 bp) were generally nucleosome-free while longer introns included one or more weakly positioned nucleosomes. The shaded area shows 95% confidence interval for the mean.

E, Metagene analysis of the plaNET-seq signal in matched introns stratified by length. Introns in both groups were chosen as pairs from the same genes to avoid any difference of transcription level. Longer introns showed a higher nascent RNAPII transcription, indicating a slower elongation compared to shorter introns. The shaded area shows 95% confidence interval for the mean.

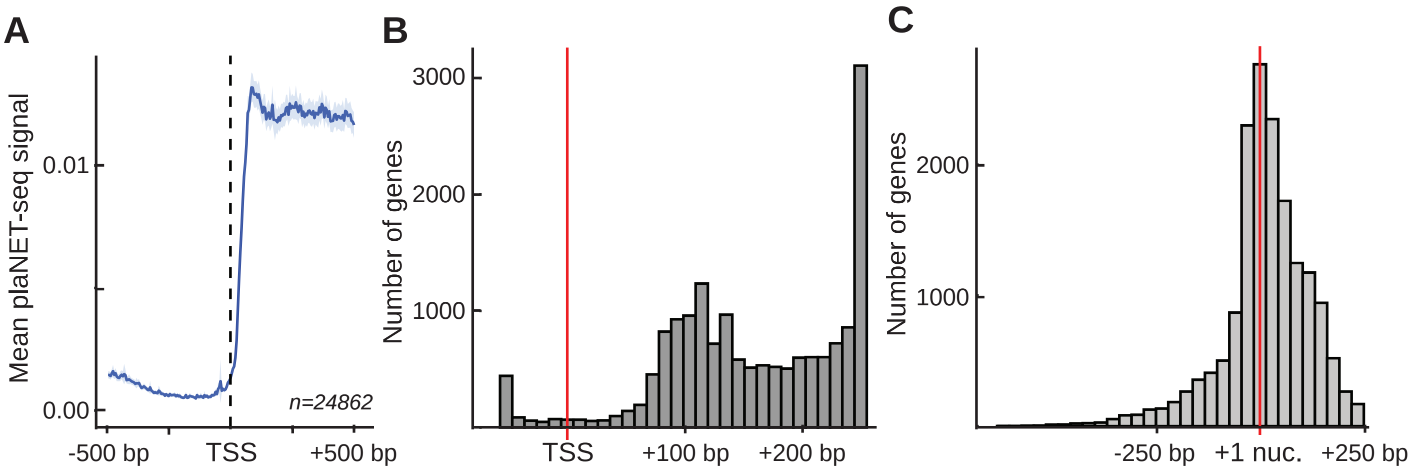

Supplementary Figure 8:
A, Metagene analysis of the plaNET-seq signal in 1 kb windows anchored at the annotated Transcription start sites (TSS) in wild type sample. No clear peak of RNAPII activity could be detected when the signal was anchored at the TSS. The shaded area shows 95% confidence interval for the mean.
B, Histogram of the distance between the TSS and the peak of promoter-proximal stalling. The median distance from the TSS was 159 bp.
C, Histogram of the distance between the center of the first nucleosome and the peak of promoter-proximal stalling. The median distance from the center of the first nucleosome was 15 bp.
